# Therapeutic Options in Docetaxel-Refractory Metastatic Castration-Resistant Prostate Cancer: A Cost-Effectiveness Analysis

**DOI:** 10.1371/journal.pone.0064275

**Published:** 2013-05-22

**Authors:** Lixian Zhong, Vickie Pon, Sandy Srinivas, Nicole Nguyen, Meghan Frear, Sherry Kwon, Cynthia Gong, Robert Malmstrom, Leslie Wilson

**Affiliations:** 1 University of California San Francisco, San Francisco, California, United States of America; 2 VA Northern California, Martinez, California, United States of America; 3 Stanford University Medical Center, Palo Alto, California, United States of America; University of Kentucky College of Medicine, United States of America

## Abstract

**Background:**

Docetaxel is an established first-line therapy to treat metastatic castration-resistant prostate cancer (mCRPC). Recently, abiraterone and cabazitaxel were approved for use after docetaxel failure, with improved survival. National Institute for Health and Clinical Excellence (NICE) preliminary recommendations were negative for both abiraterone (now positive in final recommendation) and cabazitaxel (negative in final recommendation).

**Objective:**

To evaluate the cost-effectiveness of abiraterone, cabazitaxel, mitoxantrone and prednisone for mCRPC treatment in US.

**Methods:**

A decision-tree model was constructed to compare the two mCRPC treatments versus two placebos over 18 months from a societal perspective. Chance nodes include baseline pain as a severity indicator, grade III/IV side-effects, and survival at 18 months. Probabilities, survival and health utilities were from published studies. Model cost inputs included drug treatment, side-effect management and prevention, radiation for pain, and death associated costs in 2010 US dollars.

**Results:**

Abiraterone is a cost-effective choice at $94K/QALY (quality adjusted life years) compared to placebo in our base-case analysis. Cabazitaxel and abiraterone are the most effective, yet also most expensive agents. The incremental cost-effectiveness ratios (ICER) at base-case are $101K/QALY (extended dominated) for mitoxantrone vs. placebo, $91K/QALY for abiraterone vs. mitoxantrone, $956K/QALY for cabazitaxel vs. abiraterone. Abiraterone becomes less cost-effective as its AWP increases, or if the cost of mitoxantrone side-effect management decreases. Increases in the percentage of patients with baseline pain leads to an increased ICER for both mitoxantrone and abiraterone, but mitoxantrone does relatively better. Cabazitaxel remains not cost-effective.

**Conclusion:**

Our base case model suggests that abiraterone is a cost-effective option in docetaxel-refractory mCRPC patients. Newer treatments will also need a CEA assessment compared to abiraterone.

## Introduction

Prostate cancer is the most common cancer affecting men, with 240,890 diagnosed in 2011[1]. Most patients are diagnosed with asymptomatic, clinically localized cancer; however, 10% to 20% have locally advanced or metastatic disease, treated with androgen deprivation therapy (ADT). Almost all patients become refractory to ADT [2], [3] and the disease becomes metastatic castration-resistant prostate cancer (mCRPC) for which docetaxel is the standard-of-care. Until recently, there were few FDA-approved options for patients who progressed following docetaxel treatment except for mitoxantrone, which improves quality-of-life through pain reduction, but has no demonstrated survival benefit[4]–[6].

In 2010, the chemotherapy agent cabazitaxel (Jevtana®) with prednisone was approved for treatment of mCRPC following docetaxel based on the TROPIC study, with a 15.1 month median survival compared to 12.7 months with mitoxantrone[7]. However, cabazitaxel also showed clinically significant grade III/IV neutropenia[7].

Shortly after, abiraterone (Zytiga®) with prednisone was also approved for mCRPC following a docetaxel-containing regimen. Abiraterone is a non-chemotherapeutic inhibitor of CYP17, and showed a median survival of 14.8 months compared to 10.9 months with prednisone alone. While it is mostly associated with mild grade I/II adverse events, it may lead to cardiac disorders[8] and we only included costs of grade III/IV cardiac disorders in our model, specifically dysrhythmia and cardiac arrest/ventricular fibrillation.

This rapid introduction of two new treatments for mCRPC, with a third (Enzalutamide) approved while this paper was in preparation and more in the pipeline, offers exciting new opportunities for improved survival. There are significant clinical and economic implications for optimizing these additional treatments into practice, resulting in difficult treatment decisions for patients and physicians. The UK's National Institute for Health and Clinical Excellence (NICE) is an agency of the British National Health Service (NHS) that publishes recommendations based on cost-effectiveness of drugs that are seeking approval. Their recommendations obligate the NHS to fund their use as treatment. NICE, in its final appraisal, did not recommend cabazitaxel[9], [10], and their draft evaluation decision does not recommend abiraterone either[11], leading to some controversy. This controversy was resolved with NICE reversing their draft evaluation decision for abiraterone, which is now recommended.

The purpose of this study is to evaluate the cost-effectiveness of the two new treatment options versus two placebos for patients with mCRPC following docetaxel treatment failure over 18 months from a US societal perspective.

## Methods

A decision-tree model was constructed to compare the cost-effectiveness (CE) of two treatment options: abiraterone and cabazitaxel, against two placebo groups: prednisone alone and mitoxantrone for mCRPC patients who failed docetaxel from a U.S. societal perspective. We analyzed a base-case which contains our best estimates of costs and outcomes and tested their validity with sensitivity analysis as is standard practice in cost-effectiveness analyses[12]. Our inputs and outputs are based on two published phase III multinational randomized clinical trials: TROPIC[7], comparing mitoxantrone plus prednisone to cabazitaxel plus prednisone, and COU-AA-301[8], comparing abiraterone plus prednisone to placebo which was prednisone alone. The inclusion criteria were similar in both studies: men with mCRPC and an Eastern Cooperative Oncology Group (ECOG) functional status score of < = 2 with disease progression despite prior docetaxel treatment. The main cost-effectiveness outcome is the ICER or incremental cost effectiveness ratio. The effectiveness is measured by quality-adjusted-life-years saved (QALYs). Quality adjusted life years saved is the “gold standard” outcome measurement for cost-effectiveness studies. QALYs measure the health benefits delivered by a given treatment regimen. QALYs are calculated by downwardly adjusting the life expectancy of each treatment for losses in quality of life as measured by ‘utility’. Utility is a number from 0 (indicating death) to 1 (indicating perfect health) indicting a preference for healthy compared to non-healthy health states for the time they were experienced during the time surviving.

**Figure 1:**
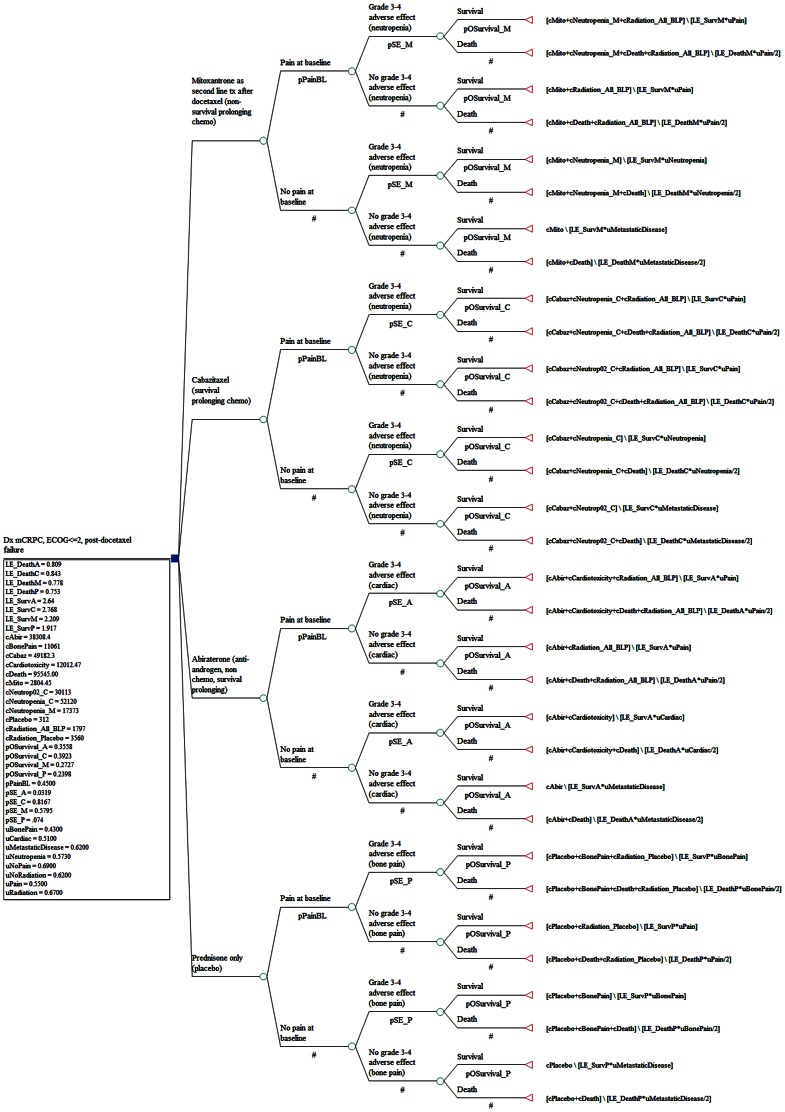
Decision Tree Structure. Decision tree model was constructed for the comparison of cost-effectiveness of two treatment options: abiraterone and cabazitaxel against two placebo ground: prednisone a lone and mitoxantrone for mCRPC patients who have failed prior docetaxel treatment. Chance node branches of our base-case analysis included baseline pain, grade III/IV side-effects, and overall survival at 18 months.

Chance node branches of our base-case analysis included baseline pain, grade III/IV side-effects, (neutropenia for cabazitaxel and mitoxantrone, severe cardiac events for abiraterone, and bone pain for prednisone alone), and overall survival at 18 months (Figure 1).

We used an 18-month time horizon with no discounting required with such a short time horizon. A cost-effective analysis should include common time horizon for accumulating costs and outcomes. We used an 18 month time horizon to standardize across the two trials. The abiraterone trial followed up survival up to 18 months while the cabazitaxel trial extended to 30 months. Based on this 18 months we modeled costs (all treatment related costs and costs of death). In addition, we modeled the life time survival based on survival rate at 18 month using a declining exponential function.

One-way and probabilistic sensitivity analyses were conducted on all variables to determine the effect of the variables on outcomes. We calculated an acceptability curve and net monetary benefits to determine the impact on decision making at different willingness-to-pay (WTP) thresholds. Generally an intervention is considered cost effective compared to an alternative intervention if the incremental cost effectiveness ratio (ICER) falls below a predetermined threshold. In the United States this threshold is a maximum of $100,000 per QALY, and we use that threshold in our analyses to determine if adoption of a treatment will add social value[13].

In addition, we analyzed a second decision model in which we used the presence or absence of baseline pain as a surrogate for disease severity. It can be helpful to clinician practicing decisions to stratify our CE analysis by some measure of disease severity. Pain was the best measure that was relatively consistent across our two clinical trials. They reported significantly different survival outcomes by their pain stratification indicating a severity of illness difference. Therefore in this secondary model we included stratified life expectancies based on the presence or absence of baseline pain from the clinical trials reported results to better model clinical sites with different proportions of severely ill patient population at docetaxel failure. In the primary base-case model we did not stratify survival by pain measures. This model is secondary because more assumptions were required in making survival estimates stratified by pain severity.

### Probabilities

In the first chance node of our base-case analysis, we divided the patient population based on presence or absence of their baseline pain as pain is an important indicator of disease severity in mCRPC patients. Pain was assessed slightly differently in the two trials. For the base-case we used the TROPIC study's 45% probability of presence of baseline pain for all treatments in order to give an additional cost for radiation treatments to those in severe pain. In the 2^nd^ chance node, we used probabilities of the most clinically relevant grade III/IV adverse events associated with different treatments; neutropenia for mitoxantrone (58%) and cabazitaxel (82%) treatments, severe cardiac events for abiraterone (4%) and grade III/IV bone pain for the prednisone alone group (7.4%) as this group didn't receive any palliative treatment. Finally, patients were assessed as deceased or alive at 18 months from the Kaplan-Meier overall survival curves [7], [8]. We chose 18 months following the COU-AA-301 trial, although the TROPIC study followed patients until 30 months, to enable comparison of survival across all four treatment groups at a consistent time point (Table 1 and Table 2).

**Table 1:** Probabilities Used in the Decision Model.

Variable	Value	Range/Distribution	Reference
**Pain at baseline –prednisone/placebo**	0.4500	(0.3375,0.5625)/Beta distribution	[7]
**Pain at baseline -mitoxantrone**	0.4500	(0.3375,0.5625)/Beta distribution	[7]
**Pain at baseline -abiraterone**	0.4500	(0.3375,0.5625)/Beta distribution	[7]
**Pain at baseline-cabazitaxel**	0.4500	(0.3375,0.5625)/Beta distribution	[7]
**Side-effects (bone pain) – Prednisone/placebo**	0.0740	(0.0555, 0.0925)/Beta distribution	[8]
**Side-effects (neutropenia) mitoxantrone**	0.5795	(0.4346, 0.7244)/Beta distribution	[7]
**Side-effects (cardiac events) abiraterone**	0.0319	(0.0239, 0.0399)/Beta distribution	[8]
**Side-effects (neutropenia) cabazitaxel**	0.8167	(0.6125, 1.0209)/Beta distribution	[7]
**Overall survival at 18 months prednisone/placebo**	0.2398	(0.1799, 0.2998)/Beta distribution	[8]
**Overall survival at 18 months |mitoxantrone**	0.2727	(0.2045, 0.3409)/Beta distribution	[7]
**Overall survival at 18 months | abiraterone**	0.3558	(0.2668, 0.4447)/Beta distribution	[8]

**Table 2:** Utilities Used in the Decision Model.

Utility Variable	Utility	Range/distribution	Utility measure	Reference
**uBonePain**	**0.43**	(0.3225, 0.5375) Beta distribution	HUI	[37]
**uCardiac**	**0.51**	(0.3825, 0.6375) Beta distribution	EQ-5D	[38]
**uPain**	**0.55**	(0.4125, 0.6875) Beta distribution	QWB	[39]
**uNeutropenia**	**0.57**	(0.4298, 0.7163) Beta distribution	SG	[40]
**uNoRadiation**	**0.62**	(0.4650, 0.7750) Beta distribution	QWB	[39]
**uMetastaticDisease**	**0.62**	(0.4650, 0.7750) Beta distribution	QWB	[39]
**uRadiation**	**0.67**	(0.5025, 0.8375) Beta distribution	QWB	[39]
**uNoPain**	**0.69**	(0.5175, 0.8625) Beta distribution	QWB	[39]

### Overall Survival and Life Expectancy (LE) Base-Case

Our cost-effectiveness outcome was incremental QALYs to calculate an incremental cost-effectiveness ratio (ICER) for each comparison beginning with the lowest cost treatment, as recommended in International Society for Pharmacoeconomics and Outcomes Research (ISPOR) guidelines [14], [15]. The primary endpoint for both clinical trials was overall survival, so the Kaplan-Meier overall survival curves and declining exponential function (DEALE) were used for our calculations of LE (Table 3). DEALE is an approximation of life expectancy by using a simple exponential function for survival, which is most accurate when survival is relatively short as it is with our mCRPC population. This function is then used to calculate life expectancy for cost-effectiveness studies. The equation is “mortality rate  = −1/t ln(S)”, where (t)  =  number of years, and (S) is fraction of subjects alive at time (t). Taking the reciprocal of this mortality rate, then gives life expectancy. This life expectancy is then adjusted downward for variations in quality of life using utilities[16], [17]. Cabazitaxel has slightly higher overall LE than abiraterone and both have higher life expectancies than mitoxantrone or prednisone alone groups (Table 3). For the base-case, we did not stratify the life expectancies based on the baseline pain. In our secondary model, we included this stratification to better reflect the effect of baseline pain on the survival benefits.

**Table 3:** Life Expectancies (LE)* (years) Used in the Decision Model**.

	Placebo	Mitoxantrone	Abiraterone	Cabazitaxel
LE_death	0.753	0.778	0.809	0.843
Range/distribution	(0.5648, 0.9413) Normal distribution	(0.5835, 0.9725) Normal distribution	(0.6068, 1.0113) Normal distribution	(0.6323, 1.0538) Normal distribution
LE_survival	1.917	2.209	2.64	2.768
Range/distribution	(1.4378,2.3963) Normal distribution	(1.6568, 2.7613) Normal distribution	(1.9800, 3.3000) Normal distribution	(2.0760, 3.4600) Normal distribution
LE overall	1.021	1.1178	1.47	1.593

* For the deceased group, we determined the LEs by calculating the area under the curve (AUC) at 18 months normalized by the percent of the deceased population [(total AUC- percent of survival at 18 months X 18 months)/percent of deceased at 18 months]. We used the Declining Exponential Approximation of Life Expectancy (DEALE) method to approximate the overall LEs of each treatment group based on survival rates at 18 months. The DEALE model is a good approximation for our study since these patients have high mortality rates which are shown to be more accurate when using the declining exponential function. **[16]**, **[17]**. The subtraction of the weighted average of the LEs of the deceased group from the overall survival at 18 months gives the weighted average of the LE of the surviving group. From there, we derived the LEs of the surviving group by dividing by proportion surviving post 18 months.

** We used the DEALE method to derive the overall life expectancies of the two subgroups differentiated by BPI score. We then extrapolated the life expectancies of the patients who died before 18 months and those remaining alive assuming a perfect declining exponential curve. Since median survival by baseline pain was only reported for abiraterone but not for the mitoxantrone or cabazitaxel groups, we assumed that all the treating groups had the same difference in their median survival between their individual subgroups with and without baseline pain (4.1 months). This was chosen as a more conservative estimate across all treatments than if we had used the abiraterone reported difference by baseline pain presence. With the overall survival derived from the each treatment groups median survival using the DEALE method, and the same difference of 4.1 months between the subgroups with baseline pain and no baseline pain for all treatments, we were able to derive the respective life expectancies of the subgroups with baseline pain and no baseline pain for each treatment group. From there, we further calculated the life expectancies of the patients who died before 18 months and who lived beyond that point for each subgroup as described previously. We then used these new life expectancies differentiated by baseline pain in our secondary decision tree analysis (results not shown).

### Costs

Treatment costs in 2010 U.S. dollars were estimated by modeling the utilization of treatment resources based on literature estimates and expert opinion of practicing physicians and pharmacists. These include mCRPC drug treatments and administration, treatment of severe side-effects (neutropenia, cardiovascular events which included dysrhythmia and cardiac arrest, bone pain treatments, radiologic treatment of severe pain, and analgesic drugs for bone pain treatment), and end-of-life hospitalizations (Table 4). Costs of physician visits, procedures and tests were from the 2010 Medicare fee schedule using Current Procedural Terminology (CPT) codes, a standard reference giving national prices[18]. Drug average whole sale prices (AWP) minus 17% for contract pricing were from the Redbook [19]. Hospitalizations and procedures costs were estimated from Healthcare Cost and Utilization Project (HCUP) national data[20]. Charges were reduced to costs using Medicare cost-to-charge ratio (0.45)[21]. 2010 costs were used because we had consistent national cost data for this year.

**Table 4:** Costs Used in the Decision Model.

Cost Variable	costs (in 2010 USD)	Range/distribution	Factors included	Reference
**Drug costs- prednisone/placebo**	$312	(156, 624) Gamma distribution	Prednisone costs are minimal. Since it is used with every treatment it's not included in the cost. Oncology doctor visits for 4 cycles*	[8], [18]
**Drug costs - mitoxantrone**	$2,804	(1402, 5609) Gamma distribution	Mitoxantrone, infusion, lab monitoring, follow-up doctor visits for 4 cycles*	[7], [18], [41]
**Drug costs- abiraterone**	$38,308	($19,154, $76,617) Gamma distribution	Abiraterone LFT monitoring (every 2 weeks for first 3 months and monthly thereafter), monthly K+ monitoring and oncology doctor visits for 8 cycles*	[8], [18], [41]
**Drug costs - cabazitaxel**	$49,182	($24,591, $98,365) Gamma distribution	Cabazitaxel, infusion, lab monitoring and follow up doctors visit for 6 cycles*	[7], [41]
**Grade III/IV neutropenia - mitoxantrone**	$17,374	($8,687, $34747) Gamma distribution	Two weeks of G-CSF treatment per cycle for half of grade III/IV neutropenia patients for 4 cycles*, one hospitalization for 9.5% of the grade III/IV neutropenia patients, one follow-up doctor's visit after discharge	[7], [23], [41], [42]
**Grade III/IV neutropenia- cabazitaxel**	$52,121	($26, 060, $104, 242) Gamma distribution	Two weeks of G-CSF treatment per cycle for all of grade III/IV neutropenia patients for 6 cycles*, one hospitalization for 19% of the grade III/IV neutropenia patients and doctor visits	[7], [18], [23], [41]–[43]
**Grade 0-II neutropenia- cabazitaxel**	$30,113	($15,066, $60226) Gamma distribution	Two weeks of G-CSF treatment per cycle for all of patients for 6 cycles* numbers	[7], [41]
**Grade III/IV cardiac disorders- abiraterone**	$12,012	($6,006, $24,025) Gamma distribution	Weighted average costs for dysrhythmia (80%) and cardiac arrest (20%) hospitalizations and 5 EKGs	[8], [18], [43]
**Grade III/IV bone pain – prednisone/placebo**	$11,061	($5,531, $22,122) Gamma distribution	Daily Bisphosphonates, morphine, Docusate Sodium, and Acetaminophen for 10.9 months**	[8], [41]
**Radiation for high baseline pain – abiraterone, mitoxantrone, cabazitaxel**	$1,797	($899, $3,594) Gamma distribution	One course of radiation therapy with 15 treatments was given to half of the patients with baseline pain in the treatment groups	[37]
**Radiation for high baseline pain- prednisone/placebo**	$3,560	($1,780, $7,120) Gamma distribution	One course of radiation therapy with 15 treatments was given to all the patients with baseline pain in the prednisone/placebo group	[37]
**Death associated hospitalization – all groups**	$95,545	($47, 773, $191, 090) Gamma distribution	Average cost of last hospitalization for severe side-effects (neutropenia and cardiac events) for an average stay of 22 days	[43], [44]

* (reported median cycle numbers were used).

** (reported medium survival for prednisone/placebo group)

Because of neutropenic deaths during cabazitaxel treatment, prophylactic G-CSF use is recommended [22]. Neutropenia related utilization includes both hospitalization costs for 19% of the patients who developed grade III/IV neutropenia [23] and 2-weeks of G-CSF (Neupogen) prophylactic treatment for 100% of patients receiving a median of 6 cabazitaxel treatment cycles [24] based on literature estimates and physician expert opinion [23], [25]. On the other hand, patients on mitoxantrone generally are minimally treated for grade III/IV neutropenia and are not treated prophylactically. We therefore applied neutropenia costs to 50% of the patients on mitoxantrone for their median 4 treatment cycles. For abiraterone, grade III/IV cardiac events costs included electrocardiograms (EKG) and hospitalizations for dysrhythmia (80%) and cardiac arrest/ventricular fibrillation (20%). Bone pain treatment was applied to patients on prednisone alone with grade III/IV bone pain. Treatments included bisphosphonates, opioids, prophylactic stool softener/stimulant combination and acetaminophen usage daily, as needed, for 18 months. Other treatment options such as denosumab are also available, and a preferred treatment by some. Our physician experts recommended that bisphosphonates were more commonly used nationally and a more conservative choice for our model[26]. There is likely significant variation in supportive care treatment nationally and more research and a treatment guideline is needed in this area.

In addition to the above costs for adverse effects, one course of 15 radiation treatments for pain palliation was given to half of those patients with baseline pain in the treatment and mitoxantrone groups (22.5% of the patients in each of these groups) and to all patients on prednisone alone with severe baseline pain (45% of the patient in the prednisone alone group). We assumed, based on clinical trial data, that cabazitaxel, mitoxantrone and abiraterone have a palliative effect which requires less pain treatment than prednisone alone. Radiation costs were estimated from published utilization information of treatments by CPT code[27]. Finally, end-of-life hospitalization costs were given to patients that died before 18 months.

### Outcomes

Utility measures patient preferences for different health states on a scale with 0 representing death and 1 perfect health. Utilities are used in cost-effectiveness analyses to adjust survival years downward for poor health states. We obtained our utility scores for different health states from the literature and the lowest score applicable to patients represented by each model branch was used to adjust survival calculations[28]–[31] (Table 2).

Quality-adjusted life years (QALYs) were calculated by multiplying corresponding utilities by life expectancies. The incremental cost-effectiveness ratio (ICER) compared each treatment option to the lowest cost option and also to the next lowest treatment option using the formula: (Cost_RX1_-Cost_RX2_)/(QALYs_RX1_-QALYs_Rx2_).

One-way sensitivity analyses and probabilistic sensitivity analyses were performed using a 1,000 iteration Monte Carlo simulation. We examined model robustness by calculating an acceptability curve where, based on a Monte Carlo simulation of all variable distributions, the number of times that a particular treatment is cost-effective given different WTP threshold levels is counted to determine the probability that each treatment is cost-effective at each WTP threshold. We also calculated net monetary benefit: NMB =  (Incremental Effect * WTP) - Incremental Cost. Any treatment with a value greater than zero is a cost-effective option compare to the next costly treatment.

## Results

Abiraterone is a cost-effective treatment compared to prednisone alone and compared to the next lowest cost option, mitoxantrone (Table 5) in our base case analysis. Prednisone alone was the lowest cost option ($75,366), mitoxantrone slightly higher ($83,171), and increasingly higher for abiraterone ($101,050) and cabazitaxel ($156,140) (Table 5). The treatment options followed the same increasing pattern for quality adjusted life expectancies, from 0.43 to 0.76 QALYs.

**Table 5:** Incremental Cost-Effectiveness Ratio (ICER)*.

treatment options	Total cost ($)	Total effect (QALYs)	incrCost ($)	incrEff (QALYs)	ICER ($/QALY)	ICER ($/LY)
**placebo**	$75,366	0.43	0	0	**0**	**0**
**mitoxantrone**	$83,171	0.51	7,805	0.08	**$100,675**	**$34,677**
**abiraterone**	$101,050	0.70	17,880	0.20	**$91,188**	**$61,182**
**cabazitaxel**	$156,140	0.76	55,089	0.06	**$955,863**	**$400,046**

* The analysis was conducted by using DATA software version TreeAge Pro 2012 (TreeAge, Williamstown, MA).

** Costs are in 2010 US dollars.

**Figure 2:**
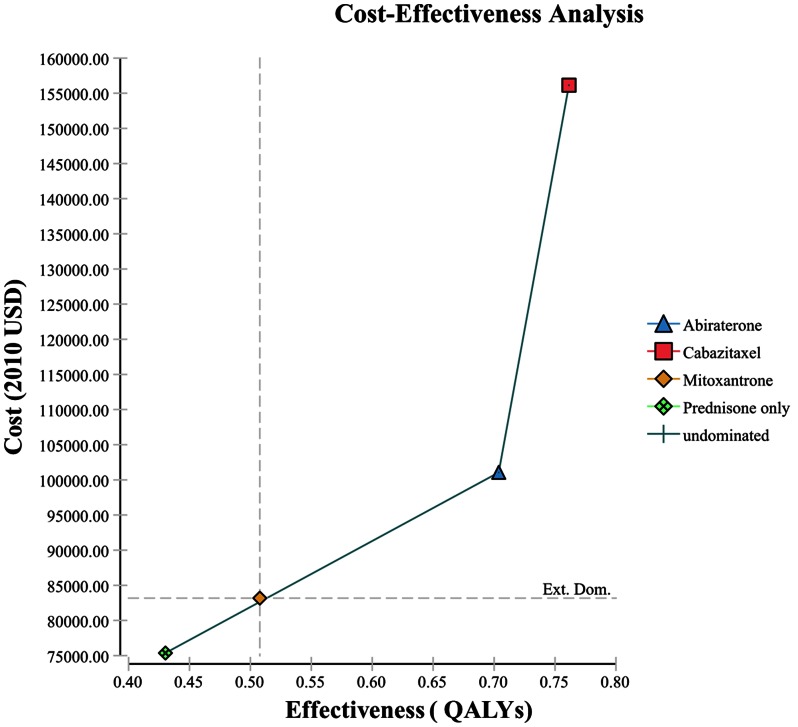
Cost-effectiveness Efficiency Frontier. *Ext. Dom. =  Extended Dominance: Mitoxantrone show extended dominance indicating that some combination of Abiraterone and Prednisone would be preferable to treating all with mitoxantrone. **Cost is in US 2010 $s; Effectiveness is in quality adjusted life years (QALYs).

The ICER for abiraterone compared to the next lowest cost option, mitoxantrone, is cost effective at $91.2K/QALY. The ICER for mitoxantrone compared to the lowest cost option, prednisone alone, is borderline cost-effective at $ 100.7K/QALY. However, the ICER for cabazitaxel compared to abiraterone is $956K/QALY which is not cost-effective, given a generally accepted WTP of $100,000. When compared with prednisone alone, abiraterone has an ICER of $94K which is still cost-effective, while the ICER of cabazitaxel compared to prednisone is $245K, still not cost-effective. Despite the slightly greater overall survival at 18 months for cabazitaxel, the additional costs associated with neutropenia treatments are so high that cabazitaxel never reaches cost-effectiveness (Table 5, Figure 2).

### Sensitivity analysis

Sensitivity analysis is used to account for the variation in treatment and other assumptions that is inherent in any CE model. We found that our model is robust to most of the variables in the decision tree but uncertainty characterizes components of nearly every CE analysis so sensitivity analysis is performed to determine the effects of variation on the final results of the analysis. Model sensitive variables in our model include variables that affect the LEs and costs of abiraterone and mitoxantrone (Table 6). For example, when the probability of survival for abiraterone (0.356; confidence interval (CI) +/− 0.089) goes below 0.348, or the probability of survival for mitoxantrone (0.273; CI +/− 0.068) goes above 0.281, the ICER for abiraterone over mitoxantrone would surpass $100,000/QALYS, making abiraterone not cost-effective over mitoxantrone. Similarly, if the LE of those surviving and treated with abiraterone (2.64; CI+/−0.66 LYs) goes below 2.56 LYs or the LE of those surviving in the mitoxantrone treated group (2.21; CI+/−0.55 LYs) goes above 2.48 LYs, the abiraterone ICER compared to mitoxantrone would exceed $100,000/QALY.

**Table 6:** Model Sensitive Variables in One Way Sensitivity Analysis.

Variable name (Base case Value)	Variable annotation	range	Mitoxantrone ICER range	Expected Value for Mitoxantrone ICER = $100K/QALY	Abiraterone ICER range	Expected Value for Abiraterone ICER = $100K/QALY
**pSE_M (0.5795)**	Probability of Side-effects (neutropenia) mitoxantrone	0.4346, 0.7244	65,414, 139,095	0.58	105,813, 77,047	0.48
**pOSurvival_A (0.3558)**	Probability of overall survival at 18 months |abiraterone	0.2668, 0.4447	100,675, 100,675	N/A*	332,303, 30,024	0.348
**pOSurvival_M (0.2727)**	Probability of overall survival at 18 months |mitoxantrone	0.2045, 0.3409	2,250,157, 8,658	0.27	42,531, 195,367	0.28
**LE_SurvA** ** **(2.64)**	Life expectancy of the patients who survived at 18 months in abiraterone treated group	1.98, 3.3	100,675, 100,675	N/A*	306,517, 67,484	2.557
**LE_SurvM (2.209)**	Life expectancy of the patients who survived at 18 months in mitoxantrone treated group	1.6568, 2.7613	initially dominated then (926,075, 47,620)	2.21	93,876, 162,982	2.32
**LE_SurvP (1.917)**	Life expectancy of the patients who survived at 18 months in prednisone treated group	1.4378, 2.3962	54,272, 694,381	1.917	91,188, 91,188	N/A*
**LE_DeathA (0.809)**	Life expectancy of the patients who died before 18 months in abiraterone treated group	0.6068, 1.0112	100,675, 100,675	N/A*	113,261, 76,315	0.717
**LE_DeathM (0.778)**	Life expectancy of the patients who died before 18 months in mitoxantrone treated group	0.5835, 0.9725	211,170, 66,092	0.778	75,557, 114,975	0.862
**LE_DeathP (0.753)**	Life expectancy of the patients who died before 18 months in prednisone treated group	0.5648, 0.9412	65,699, 215,277	0.753	91,188, 91,188	N/A*
**uNeutropenia (0.57)**	Utility for the patients who developed neutropenia side -effects	0.4298, 0.7163	210,351, 75,603	0.573	66,173, 114,868	0.634
**uMetastaticDisease (0.62)**	Utility for the patients who had metastatic disease which would apply to all patients in this study	0.465, 0.775	74,565, 154,925	0.62	138,835, 67,889	0.58
**cAbir (38,308)**	Drug costs - abiraterone	19,154, 76,617	Initially dominated then 100,674, 100,674	N/A*	23,868, 286,565	40,040
**cMito (2,804)**	Drug costs - mitoxantrone	1,402, 5,609	82,587, 136,849	2,760	98,340, 76,885	N/A*
**cNeutropenia_M(17,374)**	Grade 3/4 neutropenia - mitoxantrone	8,687, 34,747	35,745, 230,547	17,300	116,860, 39,839	14100
**cBonePain (11,061)**	Grade ¾ bone pain - placebo	5,331, 22,122	105,954, 90,117	11,700	91,188, 91,188	N/A*
**cRadiation_Placebo (3,560)**	Radiation for high baseline pain – Placebo	1,780, 7,120	111,008, 80,015	3,676	91,188, 91,188	N/A*

* N/A means that the variable did not reach the $100K WTP threshold within the plausible range tested.

** LE  =  life expectancy

In addition, abiraterone drug costs and mitoxantrone side-effect treatment costs are sensitive in our model. According to the base-case model, if abiraterone drug costs exceed $52.33 per 250 mg pill (base-case is $50) then abiraterone will no longer be cost-effective compared to mitoxantrone. Additionally, if the cost for treating grade III/IV neutropenia ($17, 374, [range: $8,687–$34,747]) in the mitoxantrone treated group falls below$14.1K, the ICER for abiraterone will go slightly above $100,000/QALY. The standard recommendation for treating neutropenia in the mitoxantrone group may vary across institutions from only 20–40% of patients treated with G-CSF vs. more. Finally, if only half of the cabazitaxel patients are given neutropenia prophylaxis, then the cost-effectiveness of cabazitaxel compared with abiraterone decreases, but still far exceeds the $100K WTP.

The dose of neupogen use for treating neutropenia or for prophylaxis may vary in practice. In our model, we used 14 days of neupogen treatment per 28-day cycle. To evaluate how the costs of different dosage of neupogen may impact our model, we also cost 10 days and 7 days of neupogen use per 28-day cycle in our model as part of the sensitivity analysis in addition to the total neutropenia costs sensitivity analysis. The reduction in neupogen dosage reduces the cost for the mitoxantrone branch and makes it a cost-effective option compared to prednisone while increasing the ICER for abiraterone compared to mitoxantrone. For the 10-day neupogen per cycle treatment regime, the ICER for mitoxantrone compared to prednisone is $79,513/QALY and the ICER for abiraterone compared to mitoxantrone is $99,555/QALY. For the 7-day neupogen per cycle treatment regime, the ICER for mitoxantrone compared to prednisone is $63,644/QALY and the ICER for abiraterone compared to mitoxantrone is $105,830/QALY. In both cases, the costs for cabazitaxel treating branch also dropped. However, it remains the most costly treatment and the ICER for cabazitaxel compared to abiraterone remained above the WTP.

Secondary Model stratified by pain as a proxy for disease severity: because we are particularly interested in reflecting differences in cost-effectiveness for populations which are more severely ill at baseline, we used presence of baseline pain as a proxy for severity and tested the cost-effectiveness when accounting for differences in overall survival by baseline pain. However, because overall survival differentiated by baseline pain was only reported in one study[32], we only included baseline pain based on overall survival difference in our secondary model. We wanted our base-case model to avoid these additional assumptions and remain more robust. For this secondary model we assumed that the differences in life expectancies between with or without baseline pain groups were the same across different treatments using the reported prednisone only group[32]. When presence of baseline pain was included with survival differences in our analysis at 45% presence of baseline pain it resulted in abiraterone dominating cabazitaxel, as the cabazitaxel survival suffered more than that of abiraterone. Also, mitoxantrone becomes more cost-effective relative to prednisone ($45.7K/QALYS). In addition, abiraterone slightly exceeds $100,000/QALY compared to mitoxantrone when baseline pain differences in survival are accounted for ($104.6K/QALYS) despite an incremental survival of 2.28 months. However, abiraterone remains cost-effective compared to prednisone alone with an ICER of $81,203/QALY and an incremental survival of 3.84 months. In this analysis, as we increase the probability of the prevalence of baseline pain (which lowers the survival benefits for all the treatment groups) it becomes less and less cost-effective to treat patients with mitoxantrone and abiraterone. The ICER for mitoxantrone compared to placebo changes from $38K/QALY to $61K/QALY and the ICER for abiraterone compared to mitoxantrone changes from $74K/QALY to $199K/QALY when the proportion with baseline pain goes from 0 to 100%. Cabazitaxel is dominated within all ranges of baseline pain prevalence.

Net monetary benefits were calculated using $100,000 WTP and showed positive values for abiraterone over mitoxantrone ($1,730) and abiraterone over placebo ($1,675) and negative values for mitoxantrone over placebo (−$55), and cabazitaxel compared to all other treatment options (<−$40,000) further supporting our ICER-based results.

**Figure 3:**
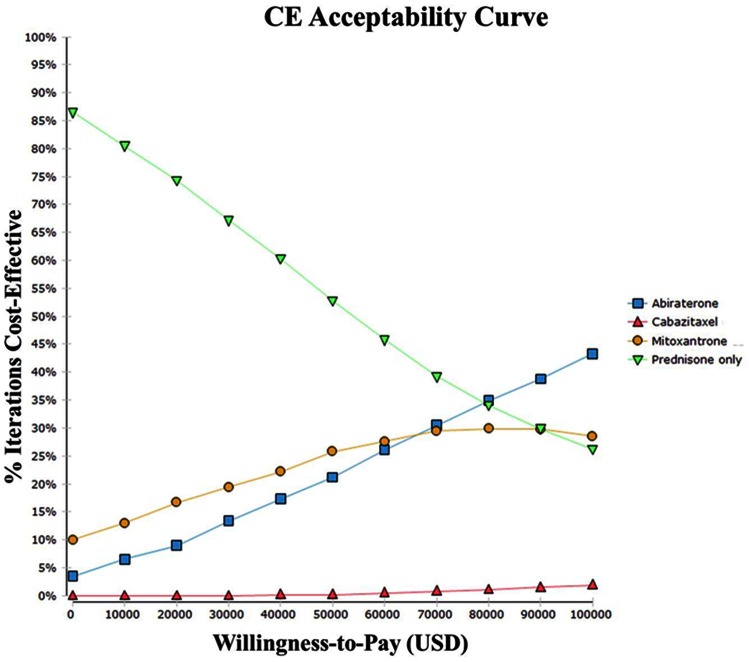
Cost-Effectiveness Acceptance Curve. Probabilistic sensitivity analysis was conducted to derive CE acceptance curve (seed: 1234).

The probabilistic sensitivity analysis for the base-case analysis showed that the probability of abiraterone being cost-effective ranges from 21.1% at WTP $50,000 to 42.0% at $100,000, and the probability of prednisone being cost-effective ranges from 52.4% at $50,000 to 25.4% at $100,000. Below a WTP of $80K, prednisone alone is most likely to be the cost-effective option. Cabazitaxel is rarely cost-effective for a WTP of $100K (4.7%) (Figure 3).

## Conclusions

We demonstrate that abiraterone treatment is cost-effective when compared to prednisone alone and to mitoxantrone. Cabazitaxel is not cost-effective in any scenarios. A recently published cost effectiveness analysis (CEA) guidance by the UK's National Institute for Health and Clinical Excellence (NICE) concluded that cabazitaxel was not cost-effective and did not recommend its use in their final appraisal [10], [32], [33]. This agrees with our findings using US treatment data and costs, although there were differences in our models and assumptions. For abiraterone the preliminary guidance decision from the NICE CEA was also negative, which may be due to their lower acceptable WTP threshold than that in US [34]. In addition, model structure, assumptions and treatment practices, especially for neutropenia and pain treatment, may differ from our studies. However, NICE also demonstrated that the cost effectiveness for abiraterone was sensitive to differences in assumptions on survival and costs, as did our model. In addition, the NICE assessment for abiraterone was controversial, given that a new treatment which both prolongs survival and avoids neutropenia may not be available to patients. Negotiations between NICE and the manufacturer following their initial decision caused a reversal and the use of abiraterone treatment in NICE's final decision.

Our US results conclude that abiraterone is cost-effective given our base-case model assumptions and costs. Although abiraterone has a slightly lower LE than cabazitaxel, it clearly has a better safety profile and cost savings from not having to treat neutropenia offsetting the cost of the drug itself. The cost-effectiveness of abiraterone is very sensitive to small changes in costs or survival. Generally, if the costs of treating neutropenia associated with mitoxantrone or abiraterone drug cost changes, the cost-effectiveness of mitoxantrone changes and that of abiraterone relative to mitoxantrone may go slightly beyond the WTP threshold. In addition, our base-case analysis demonstrates extended dominance for mitoxantrone, meaning that some combination of prednisone and abiraterone treatment would be preferable to treating all with mitoxantrone. Illness severity might be one method of stratifying patients for treatment decisions across prednisone plus pain relief, mitoxantrone, or abiraterone choices. Because of this extended dominance and borderline cost effectiveness of mitoxantrone and also the uncertainty of the costs of neutropenia treatment in mCRPC patients who fail docetaxel, it seems reasonable to treat with abiraterone until further cost evidence is obtained.

Our secondary model analysis demonstrates that, for populations that are more severely ill with lower expected survival, mitoxantrone and prednisone alone become viable choices, primarily when the majority of patients have severe baseline pain and a shortened survival. For clinical sites with severely ill patients, it may not be cost-effective to treat all patients with abiraterone, but more cost-effective to treat some with either mitoxantrone or prednisone alone. However, the lack of a survival benefit and accompanying side effects with these treatments needs consideration when making a treatment choice despite any CEA results.

Patients with mCRPC who have failed docetaxel represent one of the most challenging management issues in treating advanced prostate cancer. If 30% of the 240,890 new cases of prostate cancer diagnosed annually in US [35], [36] will be treated, then treating with mitoxantrone instead of prednisone alone would add $564 million in US costs, while using abiraterone instead of prednisone alone would cost $1.8 billion (three times higher). However, there would also be an additional benefit of 19,512 quality adjusted life years with abiraterone treatment.

Assumptions which affect costs or quality adjusted survival of mitoxantrone or abiraterone could affect our results. However, cabazitaxel was not cost-effective across a wide range of survival comparisons, thus this does not likely affect our decisions.

There is uncertainty when assigning utility to each health state, although these were the best utilities available to us and our model was fairly robust to utility changes.

In addition, our secondary model which was stratified by pain may not reflect true severity of illness. However because the clinical trials showed significant difference in survival when stratified by pain, we felt that it was an adequate surrogate of severity of illness for this cost-effectiveness comparison. The measures of pain were slightly different in the two trials as well; however this likely did not affect our results.

A cost-effectiveness study such as this one can help practicing physicians in many settings to support treatment decisions when new treatment choices are emerging and newly available. Treatment choices before the approval of first mitoxantrone and then of cabazitaxel and abiraterone, approved closely together, for castrate resistant metastatic patients who fail docetaxel treatment were to treat again with docetaxel or to forgo that and its potential severe side effects in favor of a shorter but potentially better quality of life with no active treatment. The addition of mitoxantrone allowed physicians and patients a treatment enabling them to forgo more chemotherapy and its side effect, but gain a pain reduction benefit, even without proven survival efficacy. This choice could be made by those not wishing to stop treatment but wishing to stop aggressive treatment and side effect risks.

Next, with the introduction of two additional drugs, both offering a survival benefit, a new choice was introduced; first cabazitaxel which added significant survival but still with the risk of neutropenia, and second abiraterone which also added significant survival benefit (arguably a bit less than cabazitaxel), but with no risk of neutropenia.

As these new drugs come on the market, physicians and patients are sorting out the risks and benefits on an individual basis and the medical groups and insurers wish to know if the added benefit is worth the costs. This cost-effectiveness study addresses that question of cost-effectiveness across all of these treatments to assist decision making when weighing costs and benefits. In addition, our study begins to hint at the decision making for those at different illness severity (defined by pain score) to see how the cost-effectiveness changes for those who are severely ill and have the lower end of the survival benefit shown in the clinical trials. Our data demonstrate that abiraterone is the most cost-effective choice and that this choice begins to change in favor of mitoxantrone as the survival benefit decreases in the highest pain group of mCRPC. Treatment choices should first be made on the basis of efficacy studies, but cost-effectiveness can assist decision making when weighing the comparative value of drugs.

The main limitation for our study and those conducted by NICE is due to the considerable uncertainty about the extent and costs of prophylaxis and treatment of neutropenia for the mitoxantrone and cabazitaxel treated patients and of pain treatment [22], [24]. More data is needed on these costs because our model is sensitive to all the costs in the model.

Abiraterone is currently the cost-effective choice in our base-case analysis. As the new drugs specific to docetaxel-refractory mCRPC continue to become available, treatment algorithms to mCRPC will likely evolve. Since this study was completed, newer agents have entered the market, and this CE model can be used to re-examine the cost-effectiveness of all these different treatments.
